# Streaming Potential Experiment on Sandstone Core Samples Based on Current Source Model under Different Sodium Chloride Solutions

**DOI:** 10.3390/s24113514

**Published:** 2024-05-29

**Authors:** Chenggang Yin, Wei Guan, Hengshan Hu

**Affiliations:** 1 College of Intelligent Manufacture, Taizhou University, Taizhou 317000, China; 2 Department of Astronautics and Mechanics, Harbin Institute of Technology, Harbin 150001, China; guanw@hit.edu.cn (W.G.); hhs@hit.edu.cn (H.H.)

**Keywords:** core sample, electrokinetic effects, streaming potential, circuit model, streaming current coefficient

## Abstract

The streaming potential effect has a wide range of applications in geophysics. The core streaming potential experiment requires that there is no external circuit at both ends of the core, but a measurement circuit must be introduced to measure the voltage between both ends of the core which will cause an external circuit. In order to analyze the effect of measurement circuits on the streaming potential experiment, this paper proposes a core current source model, i.e., the core in the streaming potential experiment is regarded as a circuit composed of a current source whose output current is equal to the seepage current and the core resistance. By changing the resistance value of the external circuit, it is found that the seepage current is not affected by the external resistance but by the excitation pressure. Experiments on the streaming potential of 20 sandstone cores under distilled water, 0.01 mol/L, 0.02 mol/L, 0.05 mol/L, 0.1 mol/L, 0.2 mol/L, 0.4 mol/L, and 0.6 mol/L sodium chloride solutions revealed that the effect of the external circuit on the streaming potential signal increased with decreasing mineralization. For distilled water-saturated sandstone cores, the effect of the external circuit was about 2%.

## 1. Introduction

The solid–liquid interface of a fluid-containing pore medium selectively adsorbs some ions through covalent bond breaking and ion exchange, leaving a net surplus of mobile ions in the pore channel, which in turn forms a double electric layer structure near the solid–liquid interface [1]. By closing the sides of a cylindrical core and applying an excitation pressure to its core end face, the fluid containing net surplus ions in the core pores will flow in the direction of the decreasing pressure gradient, thus generating a seepage current. The accumulation of charge on the core end face produces an electric field opposite to the seepage current, which in turn produces a conduction current opposite to the seepage current. If no external circuit exists, then the seepage current is equal and opposite to the conduction current. The potential difference between the two end faces of the core at this point is called the streaming potential. The ratio of the streaming potential to the excitation pressure is called the streaming potential coefficient [2,3]. This phenomenon of the excitation current of fluid flow in the pore channel of the pore medium caused by the double electric layer is called the streaming potential effect [4].

The streaming potential is affected by mineralization [5], temperature [6], pore size distribution [7], solution saturation [8], solution viscosity [9], etc. It can be used in the fields of seismic early warning [10], kinetic drilling and logging [11], the monitoring of polymer [12] or brine-driven oil extraction [13], the monitoring of volcanoes [14], the monitoring of underground glaciers [15], the monitoring of the thickness of the permafrost layer under the Qinghai–Tibet Railway [16], the monitoring of the mining of submarine hydrothermal deposits [17], assessing the landfill capacity of underground carbon dioxide [18,19], and evaporative solar power generation [20].

Core streaming potential experiments are an important way to obtain the streaming potential. Ahmad [5] used a unidirectional direct current (DC) pressure source to excite the streaming potential of sand in a resin tube. The unidirectional DC pressure source tends to clog the orifice and fails to suppress the zero drift and low-frequency noise of the streaming potential signal. Jouniaux & Pozzi [21] used a reciprocating DC pressure source to excite the streaming potential of sandstone, which better suppressed the clogging of the orifice and the zero drift, but there was still the interference of the low-frequency noise. Revil et al. [22] used a single-ended alternating current (AC) pressure source to excite the streaming potential of volcanic rocks, which further suppressed the interference of the low-frequency noise, but the AC pressure source directly contacted the core gripper, resulting in a significant resonance of the core gripper, which in turn affected the streaming potential measurement. Yin C. et al. [23] used a double-ended AC pressure source to excite the streaming potential of sandstone and artificial rock samples. The double-ended AC pressure source does not directly contact the core gripper, thus eliminating the effect of resonance. Luong & Sprik [24] have carried out streaming potential measurements as a function of electrolyte concentration and temperature for a set of well-defined consolidated samples. Cherubini A. et al. [25] measured the streaming potential coupling coefficient of natural saturated and unsaturated carbonate rocks.

The current core streaming potential experiments have the following problems: theoretically, the acquisition of the streaming potential requires that the core is isolated in the circuit, but in practice, it is necessary to apply the measurement circuit at both ends of the core to acquire the voltage at both ends of the core. Therefore, it is expected to model the circuit of the core, analyze the effect of the measurement circuit on the streaming potential, and give a correction formula.

## 2. Experimental Methods

### 2.1. Core Current Source Model

From the theory of streaming potential, it is known that there are two kinds of currents in the pore channel of rock samples, one is the seepage current caused by the movement of net surplus cations, and the other is the conduction current obeying Ohm’s law. The rock sample current source model considers that the seepage current is determined by the excitation pressure as well as the characteristics of the pore medium itself, independent of the external circuit, and can be regarded as a constant current source when analyzing the influence of the external circuit.

**Figure 1:**
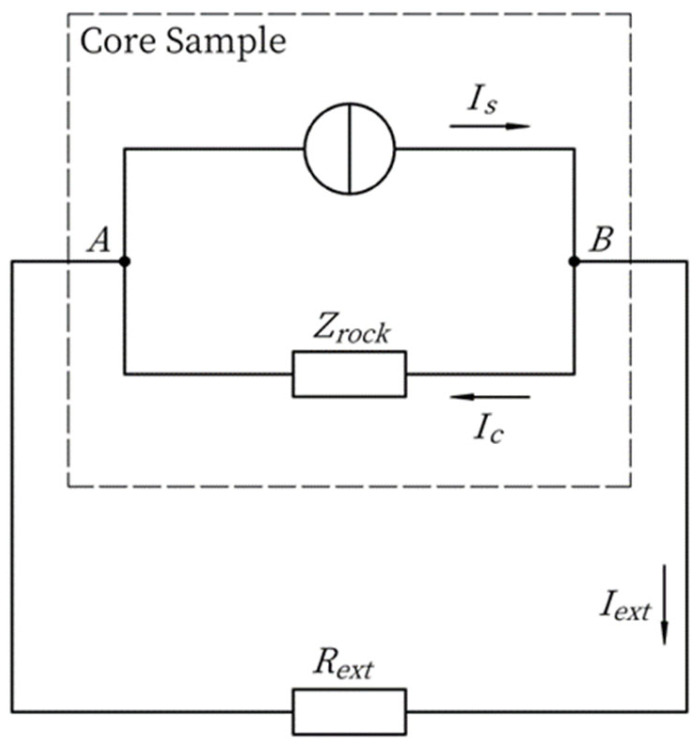
The current source model of the core sample in the streaming potential experiment.

The schematic diagram of the rock sample current source model is shown in Figure 1, in which A and B indicate the two end faces of the rock sample, the dashed box is the circuit model of the rock sample, and the undashed box is the external circuit, which is the external resistance. Inside the rock sample, the seepage current is not affected by the external circuit, while the conduction current is affected by the external circuit; the rock sample impedance is determined by the mineralization in the pore channel and the geometrical special rows of the pore medium.

(1) $$Z_{core}=\frac{l}{\sigma_{r}\phi A},$$

where $Z_{core}$ is the rock sample impedance, l is the length of the core, $\sigma_{r}$ is the electric conductivity of the core, ϕ is the porosity of the core, and A is the cross-sectional area of the core.

From Kirchhoff’s current law,

(2) $$I_{s}=I_{c}+I_{ext},$$

where $I_{s}$ is the seepage current, $I_{ext}$ is the external current, and $I_{c}$ is the conduction current. From Ohm’s law, it follows that

(3) $$\Delta U_{AB}=I_{c}Z_{core},$$

where $\Delta U_{AB}$ is the potential difference between the two ends of the rock sample

(4) $$\Delta U_{AB}=I_{ext}R_{ext},$$

where $R_{ext}$ is the total external resistance between the two ends of the rock sample. Substituting Equations (3) and (4) into Equation (2) gives

(5) $$I_{s}=\Delta U_{AB}\left(\frac{1}{Z_{core}}+\frac{1}{R_{ext}}\right),$$

### 2.2. Experimental Verification of the Current Source Model

In order to verify the rock sample current source model of the streaming potential experiment, an adjustable resistance box has been applied at both ends of the rock sample, and then the potential difference between the two ends of the rock sample has been measured. The resistance value of the resistance box ZX21 is 0.1 Ω to 0.1 MΩ, and its resistance value is calibrated by a multimeter Fluke 8846A before use; the rated input impedance of the data acquisition card NI PXI-5922 is 1 MΩ. The resistor box and the data acquisition card are connected in parallel, and their total external resistance is

(6) $$R_{ext}=(R_{var}R_{DAC})/(R_{var}{+R}_{DAC}),$$

where $R_{var}$ is the resistance value of the resistor box and $R_{DAC}$ is the input impedance of the data acquisition card NI PXI-5922.

The rock sample impedance $Z_{core}$ is measured experimentally by the conductivity of the rock sample. After measuring the external resistance $R_{ext}$, the rock sample impedance $Z_{core}$ and the potential difference $\Delta U_{AB}$ between the two ends of the rock sample, and the seepage current $I_{s}$ of the rock sample can be obtained through Equation (5).

**Figure 2:**
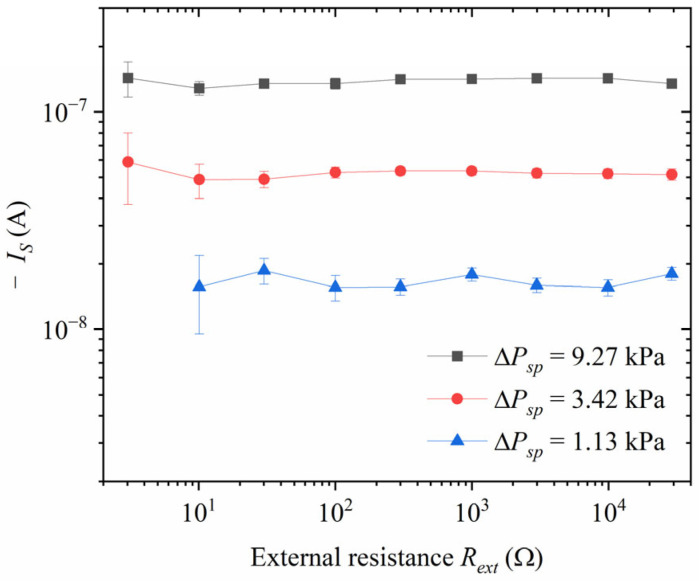
The streaming currents with varying external resistance in the streaming potential experiment.

The measurements of the rock sample current source model for the streaming potential experiment are shown in Figure 2, where the sample is sandstone S02 (as shown in Table 1) with a mineralization of 0.4 mol/L, the excitation frequency is 1 Hz, and the excitation pressures are 9.27 kPa (squares), 3.42 kPa (dots), and 1.13 kPa (upper triangles), respectively. The horizontal coordinate of the graph is the external resistance obtained by paralleling the resistance value of the resistor box and the input impedance of the data acquisition card, which ranges from 3.04 to 29.1 kΩ; the vertical coordinate is the absolute value of the seepage current (a negative number). As seen in Figure 2, the absolute value of the seepage current is positively correlated with the excitation pressure and independent of the external resistance. In other words, when the parameters of the rock sample itself are kept constant, the seepage current can be regarded as the output current of a constant current source controlled by the excitation pressure and independent of the external circuit. This is consistent with the assumptions of the rock sample current source model, thus proving that the rock sample current source model is correct. 

### 2.3. Correction of Experimental Errors

When the external circuit is disconnected, substituting into Equation (2) yields

(7) $$I_{s}=I_{c}{|}_{I_{ext}=0}$$

Substituting Equation (7) into Equation (3) gives

(8) $$\Delta U_{AB}{|}_{I_{ext}=0}=I_{s}Z_{core}$$

When $I_{ext}=0\;and$ $\Delta U_{sp}=\Delta U_{AB}$ and therefore

(9) $$\Delta U_{sp}=I_{s}Z_{core},$$

where $\Delta U_{sp}$ is the streaming potential.

Substituting Equation (5) into Equation (8) gives

(10) $$\Delta U_{sp}=(1+\frac{Z_{core}}{R_{ext}})\Delta U_{AB},$$

It can be seen that when the shunt effect of the external resistance is not negligible, the measured value $\Delta U_{AB}$ of the streaming potential has a certain deviation from the actual streaming potential $\Delta U_{sp}$, and the relative error of the streaming potential $\delta_{sp}$ is

(11) $$\delta_{sp}=\frac{\Delta U_{AB}-\Delta U_{sp}}{\Delta U_{sp}}=-\frac{Z_{core}}{R_{ext}+Z_{core}},$$

as can be seen from Equation (11). Only when $R_{ext}>>Z_{core}$, the effect of the external circuit can be neglected. In fact, for streaming potential experiments with low mineralization, the measured value $\Delta U_{AB}$ of the streaming potential needs to be corrected by Equation (10).

In the streaming potential experiment, the external circuit at both ends of the rock sample contains only the data acquisition card PXI-5922. The external resistance of the rock sample current source model is equal to the input impedance of the data acquisition card, namely,

(12) $$R_{ext}=R_{DAC}=1\;M\Omega ,$$

where $R_{DAC}$ is the input impedance of the data acquisition card.

The samples in this study consist of 20 sandstones, where the sandstone samples were intercepted from rock samples obtained from drilling. These samples are cylindrical with a diameter of 2.5 cm, provided by China National Petroleum Group Logging Company Limited, and their physical parameters are shown in Table 1. Among them, particle density is the density of the solid-phase matrix of the rock samples; porosity is the ratio of the volume of internal pores to the total volume of the rock samples; and gas permeability is the permeability of the rock samples measured by Darcy’s law pressure-fall method based on the use of gas as a medium.

**Figure 3:**
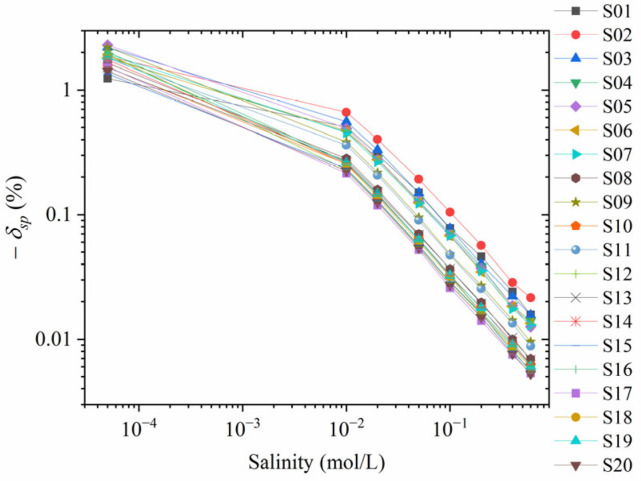
The relative error of the streaming potential with different salinities in the streaming potential experiment when the circuit model is not considered.

The impedance $Z_{core}$ of the rock sample can be measured by the conductivity experiment of the rock sample, which is affected by the mineralization $C_{f}$ of the solution within the pores of the rock sample as well as the surface conductance of the rock sample. The measurement error $\delta_{sp}$ in the streaming potential can be obtained by substituting the external resistance $R_{ext}$ and rock sample impedance $Z_{core}$ into Equation (11), as shown in Figure 3. The measurement error $\delta_{sp}<0$ of the streaming potential is due to the fact that the external resistance causes the measurement of the streaming potential to become smaller. In order to use logarithmic coordinates for the measurement error, the vertical coordinates in Figure 3 are the absolute values of the measurement error.

As seen in Figure 3, the smaller the mineralization of the saturated fluid of the rock sample, the larger the measurement error $\delta_{sp}$ of the streaming potential. The corresponding distilled water-saturated sandstone has a measurement error of about 2%. At this point, a correction must be made by Equation (11). In contrast, for the samples with a mineralization of 0.05 and above, the absolute value of the measurement error is ≤0.2% and no error correction is required.

## 3. Experimental Results and Analysis

### 3.1. Sample Pre-Treatment

The rock samples need to be pre-treated before use, including cleaning, drying, solution preparation, and solution saturation, as shown below: Oil and salt washing using a Soxhlet extractor, in which the solvent for oil washing is carbon tetrachloride, and the solvent for salt washing is a benzene–methanol solution with a volume ratio of 1:3. The strength of the kinetic–electrical coupling signal and the state of charge distribution within the pores are closely related to the mineralization of the fluid within the pores, and the accuracy of the fluid mineralization within the pores of the rock samples relies on the cleaning quality of the rock samples. Therefore, the thorough cleaning of the residual NaCl solution in the pores of the rock samples is one of the keys to accurately analyzing the effect of the kinetic–electric coupling. The basis for determining whether the cleaning is thorough or not is to take out the solution in the Soxhlet extractor bucket and use silver nitrate to check whether the solution contains chloride ions. In addition, when the solution in the Soxhlet extractor barrel is siphoned back into the flask, the solution in the flask stops boiling because of the temperature drop. We found that after many times of siphoning to stop boiling, the flask of the booster will gradually fail, if not in the old booster before the failure of timely replenishment of the new booster, the solution in the flask will be boiled, thus affecting the normal cleaning work. To prevent the solution inside the flask from boiling, we added an aiding agent every two hours. The drying process was conducted in two stages. Firstly, the cleaned rock samples were placed in a ventilated area to dry naturally. After that, they were transferred to an oven for further drying. We used an electronic balance and a volumetric flask to prepare an analytically pure sodium chloride reagent and deionized water into a specified concentration of NaCl solution. A nylon membrane with a pore size of 0.45 μm and a sand-core filter was employed to filter the NaCl solution under negative pressure, effectively removing trace insoluble impurities carried by the sodium chloride reagent. The filtered solution was then transferred to an extraction bottle and subjected to a vacuum of −0.08 MPa (equivalent to the saturated vapor pressure of water at 60 °C). This step was intended to reduce the amount of air dissolved in the NaCl solution, lowering its saturation level. Finally, the solution was sealed and stored in an appropriate container.

In this paper, we need to measure different mineralization degrees of NaCl solution-saturated rock sample experiment. The higher the mineralization at the time of measurement, the higher the tolerance for the residual NaCl before measurement, and the more difficult to clean after measurement. Therefore, in this paper, measurements were taken in order from low to high mineralization (distilled water, 0.01 mol/L, 0.02 mol/L, 0.05 mol/L, 0.1 mol/L, 0.2 mol/L, 0.4 mol/L, and 0.6 mol/L sodium chloride solutions). The saturation of the rock samples in the solutions was performed using a vacuum-pressurized saturation device. The rock samples were first placed in a pressure-resistant container, and then the container was evacuated for 4 to 6 h, followed by filling the container with the freshly made solutions of specified mineralization, and then applying a pressure of 20 MPa to the container for 2 to 3 h, and finally relieving the pressure and removing the rock samples. The pre-treated rock samples were stored in airtight containers containing the solution.

### 3.2. Experimental Signals

The signals acquired in this paper are continuous period signals. By adjusting the excitation frequency, sampling frequency, and sampling time, the measured signal is acquired for an integer number of cycles, and an integer number of points are acquired in each cycle. The unit of sampling frequency is S/s (samples per second, i.e., the number of samples per second), which is limited by the acquisition card and cannot be changed arbitrarily. For the data acquisition card NI PXI-5922, its sampling frequency at the highest resolution (24bit) is 50 kS/s or 500 kS/s. In this paper, we use the sampling frequency of 50 kS/s to reduce the size of the data packet. For ultra-low-frequency signals, the packet size obtained with a 50 kS/s sampling frequency is still too large, and it is meaningless to have too many sampling points in one cycle. In order to compress the measurement data, for the signals of 2 Hz and below, this paper performs an arithmetic average of every 1000 sampling points obtained, so that an equivalent 50 S/s sampling frequency can be obtained; for signals above 2 Hz, this paper does not perform an average of the obtained data.

**Figure 4:**
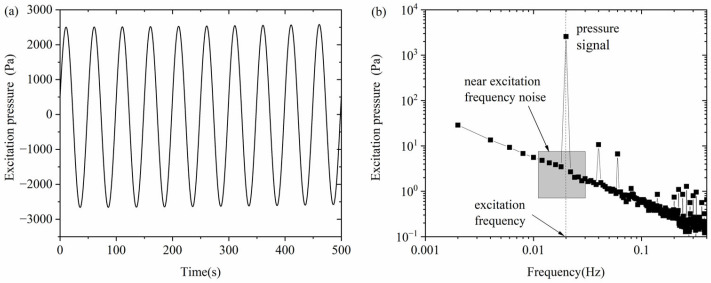
A measured signal of the excitation pressure. (**a**) Time domain; (**b**) frequency domain.

Figure 4 shows the measured signal of the excitation pressure of a streaming potential experiment, in which the rock sample number is S06, the mineralization is 0.4 mol/L, the excitation frequency is 0.02 Hz, and a total of 10 cycles of data were collected. As shown in Figure 4a, the time-domain signal of the excitation pressure is an approximately pure sinusoidal signal. As shown in Figure 4b, the frequency domain signal of the excitation pressure includes the excitation pressure signal at the excitation frequency, the harmonic signals at the integer multiples of the excitation frequency, and other background noise. The peak excitation pressure can be obtained by directly reading the 10th (number of cycles acquired) datum of the frequency domain signal. In this paper, the error estimation of the measured signal is dependent on the estimation of the noise at the excitation frequency. Although noise rises and falls randomly, it is frequency-dependent, which means the excitation frequency noise has a kind of correlation with the noise that is near the excitation frequency. As shown in Figure 4b, several noises near the excitation frequency of 0.02 Hz have been extracted and compared. The maximum one is used to estimate the noise at the excitation frequency which is about 5 Pa at this time, and thus the excitation pressure $\Delta P_{sp}=2578\pm 5\;\mathrm{Pa}$.

### 3.3. The Relationship between Streaming Current Coefficient and Permeability

The streaming potential is affected by the excitation pressure. Thus, the streaming potential coefficient and the streaming current coefficient are defined [5,26].

(13) $$C_{SP}=-\frac{∆U_{SP}}{∆P_{SP}},$$

where $C_{SP}$ is the streaming potential coefficient, the unit is $V/P_{a}$, and $∆P_{SP}$ is the excitation pressure.

(14) $$C_{SC}=-\frac{∆U_{SP}}{{∆P_{SP}Z}_{core}},$$

where $C_{SC}$ is the streaming current coefficient and the unit is $A/P_{a}$. As shown in Equations (13) and (14), the determination of both the streaming potential coefficient and the streaming current coefficient depends on the measurement of the streaming potential.

With Equations (9) and (14), the streaming current coefficient can be expressed in a more concise way as follows:

(15) $$C_{SC}=-\frac{I_{S}}{∆P_{SP}},$$

Equation (14) is applicable to experimental measurements, while Equation (15) is applicable to data analysis.

**Figure 5:**
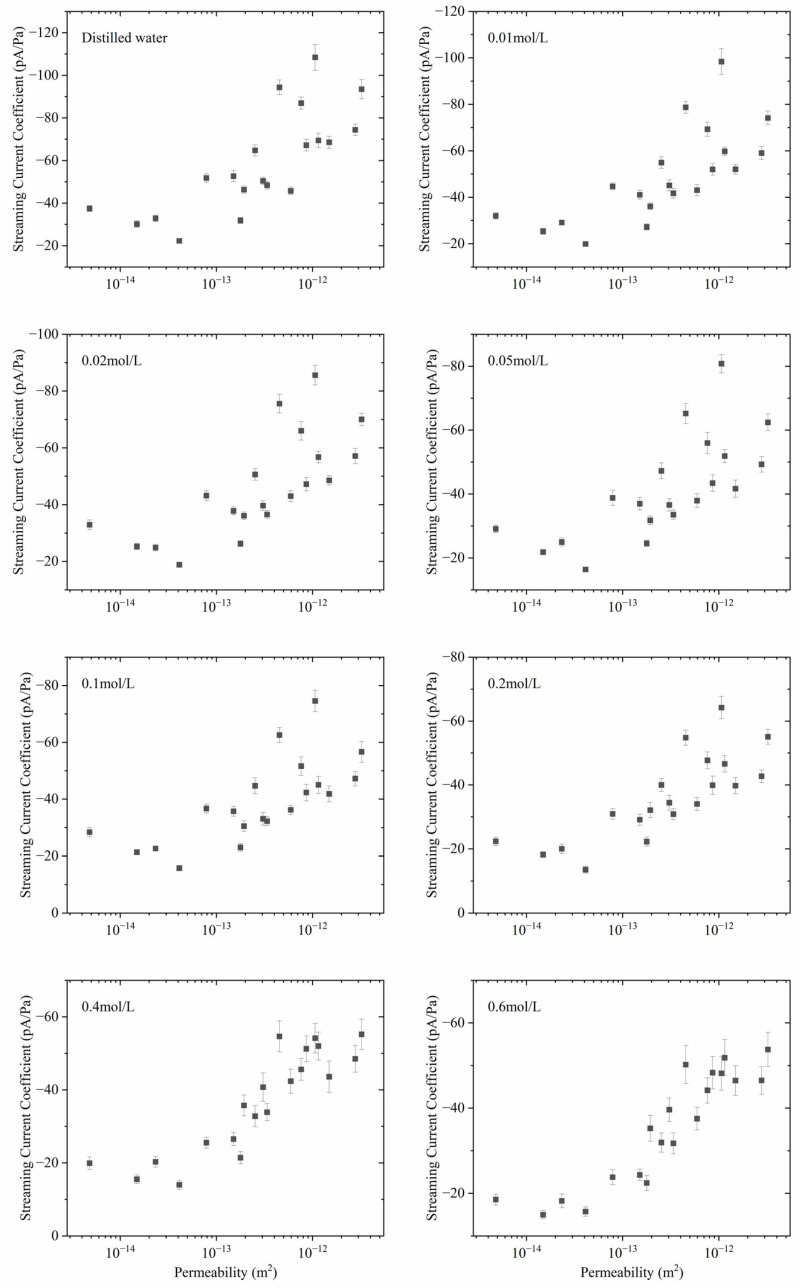
The streaming current coefficient with the varying permeability of the sandstone samples in different salinities.

The relationship between the streaming current coefficients with permeability for the sandstone rock samples is shown in Figure 5, respectively. The letter p before the unit $A/P_{a}$ means *pico*-. The absolute value of the streaming current coefficient is positively correlated with the permeability. With the same excitation pressure, when the permeability increases, the seepage velocity will increase, and then the seepage current will increase. So, the absolute streaming current coefficient, which is the ratio of the seepage current and the excitation pressure, is positively correlated with the permeability. The streaming current coefficient decreases slowly with increasing salinity. The streaming current coefficient under distilled water is about half that under 0.6 mol/L salinity. Given that the salinity varies over several orders of magnitude, the change in the streaming current coefficient is relatively small. This implies that salinity has some effect on the number or distribution of the net surplus of mobile ions in the pore of the sample, but it is not large.

### 3.4. The Relationship between Streaming Current Coefficient and Porosity

**Figure 6:**
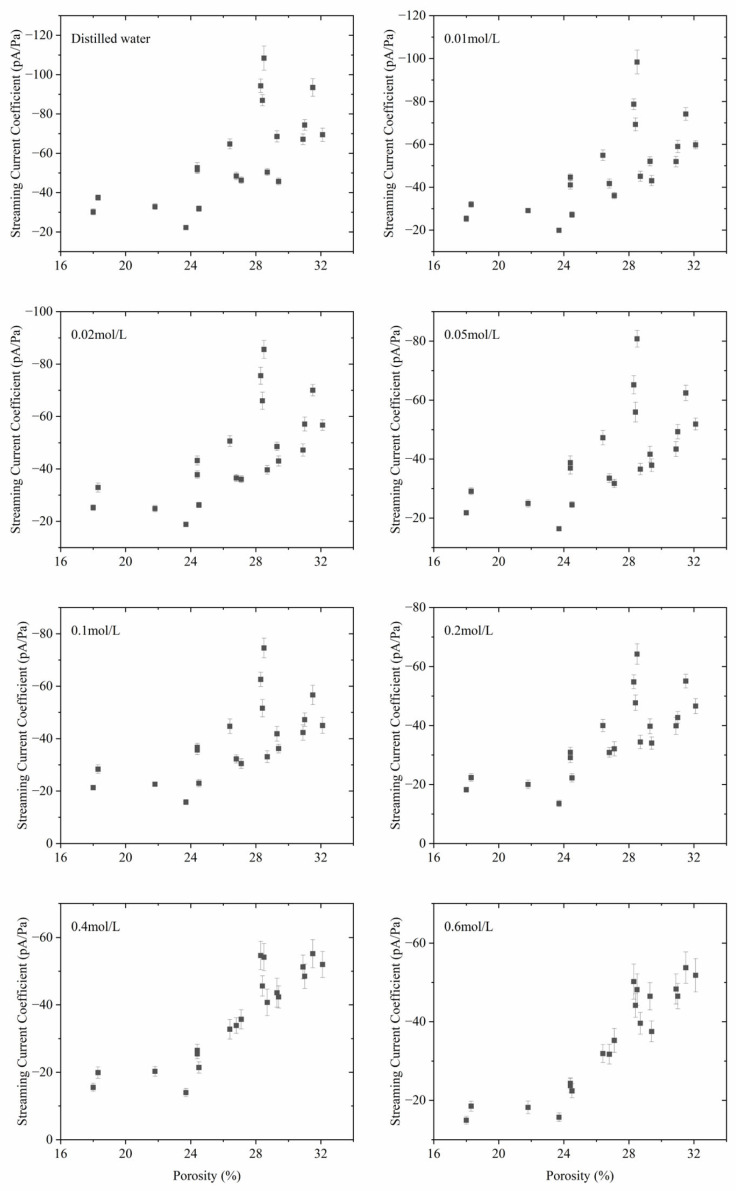
The streaming current coefficient with the varying porosity of the sandstone samples in different salinities.

The variation relationship of the current potential coefficient with the porosity of the rock samples is shown in Figure 6. The absolute value of the streaming current coefficient increases with the increase in porosity. The slope of the streaming current coefficient to porosity is steeper when porosity is higher, and vice versa. Furthermore, with increasing salinity, the data points are more densely distributed, which means the correlation between the streaming current coefficient and porosity is enhanced in high salinity. It can be seen that the correlation between the streaming current coefficient and the permeability is almost the same as the correlation between the streaming current coefficient and the porosity, and the correlation of the latter is slightly better than that of the former under 0.4 mol/L and 0.6 mol/L salinities.

## 4. Conclusions

In this paper, the current source model of rock core is established for the streaming potential experiment of rock core, and the following conclusions are obtained.

Based on the mechanism of the streaming potential effect, the rock sample current source model for the streaming potential experiment is proposed, and the model is verified through experiments, and then the systematic error caused by the measurement circuit is eliminated through the model. The current source model of the rock samples uses the parallel seepage current and the impedance of the rock samples to represent the internal circuit of the rock samples, and considers that the amplitude of the seepage current is only affected by the amplitude of the excitation pressure and the characteristics of the pore medium itself, and has nothing to do with the parameters of the external circuit. The experiments show that by changing the resistance of the external circuit, the voltage at both ends of the rock samples will also be changed, which proves that the resistance of the external circuit affects the measurement of the streaming potential signal; changing the resistance of the external circuit, the amplitude of the voltage at both ends of the rock samples is consistent with that predicted by the model of the rock current source, which proves that the model of the current source of the rock samples is correct. Considering the actual measurement circuit it is found that, for the sandstones, the relative error of the system is about 2%.

## Figures and Tables

**Table 1:** Parameters of sandstone samples.

Number	Type	Density g/cm^3^	Porosity %	Permeability ×10^−15^ m^2^
S01	Grayish-white fine sandstone	2.62	18.3	4.8
S02	Grayish-white fine sandstone	2.68	18.0	14.9
S03	Grayish-white fine sandstone	2.67	21.8	23.3
S04	Gray fine sandstone	2.67	23.7	41.2
S05	Grayish-white medium sandstone	2.64	24.4	78.7
S06	Grayish-white fine sandstone	2.67	24.4	151
S07	Grayish-white fine sandstone	2.67	24.5	178
S08	Grayish-white medium sandstone	2.61	27.1	194
S09	Grayish-white medium sandstone	2.63	26.4	253
S10	Grayish-white medium sandstone	2.63	28.7	306
S11	Grayish-white medium sandstone	2.69	26.8	337
S12	Grayish-white medium sandstone	2.65	28.3	453
S13	Grayish-white coarse sandstone	2.65	29.4	594
S14	Grayish-white coarse sandstone	2.62	28.4	762
S15	Gray coarse sandstone	2.63	30.9	862
S16	Gray coarse sandstone	2.61	28.5	1066
S17	Grayish-white coarse sandstone	2.61	32.1	1152
S18	Grayish-white medium sandstone	2.65	29.3	1491
S19	Grayish-brown coarse sandstone	2.61	31.0	2785
S20	Grayish-brown coarse sandstone	2.62	31.5	3241

## Data Availability

The datasets used and/or analyzed during the current study are available from the authors upon reasonable request.
